# Bardoxolone Methyl Displays Detrimental Effects on Endothelial Bioenergetics, Suppresses Endothelial ET-1 Release, and Increases Endothelial Permeability in Human Microvascular Endothelium

**DOI:** 10.1155/2020/4678252

**Published:** 2020-10-14

**Authors:** Ewa Szczesny-Malysiak, Marta Stojak, Roberto Campagna, Marek Grosicki, Marek Jamrozik, Patrycja Kaczara, Stefan Chlopicki

**Affiliations:** ^1^Jagiellonian Centre for Experimental Therapeutics (JCET), Jagiellonian University, Bobrzynskiego 14, 30-348 Krakow, Poland; ^2^Department of Clinical Sciences, Polytechnic University of Marche, Via Ranieri 65, 60131 Ancona, Italy; ^3^Chair of Pharmacology, Jagiellonian University Medical College, Grzegorzecka 16, Krakow, Poland

## Abstract

Nrf2 is a master regulator of antioxidant cellular defence, and agents activating the Nrf2 pathway have been tested in various diseases. However, unexpected side effects of cardiovascular nature reported for bardoxolone methyl in patients with type 2 diabetes mellitus and stage 4 chronic kidney disease (the BEACON trial) still have not been fully explained. Here, we aimed to characterize the effects of bardoxolone methyl compared with other Nrf2 activators—dimethyl fumarate and L-sulforaphane—on human microvascular endothelium. Endothelial toxicity, bioenergetics, mitochondrial membrane potential, endothelin-1 (ET-1) release, endothelial permeability, Nrf2 expression, and ROS production were assessed in human microvascular endothelial cells (HMEC-1) incubated for 3 and 24 hours with 100 nM–5 *μ*M of either bardoxolone methyl, dimethyl fumarate, or L-sulforaphane. Three-hour incubation with bardoxolone methyl (100 nM–5 *μ*M), although not toxic to endothelial cells, significantly affected endothelial bioenergetics by decreasing mitochondrial membrane potential (concentrations ≥ 3 *μ*M), decreasing spare respiratory capacity (concentrations ≥ 1 *μ*M), and increasing proton leak (concentrations ≥ 500 nM), while dimethyl fumarate and L-sulforaphane did not exert such actions. Bardoxolone methyl at concentrations ≥ 3 *μ*M also decreased cellular viability and induced necrosis and apoptosis in the endothelium upon 24-hour incubation. In turn, endothelin-1 decreased permeability in endothelial cells in picomolar range, while bardoxolone methyl decreased ET-1 release and increased endothelial permeability even after short-term (3 hours) incubation. In conclusion, despite that all three Nrf2 activators exerted some beneficial effects on the endothelium, as evidenced by a decrease in ROS production, bardoxolone methyl, the most potent Nrf2 activator among the tested compounds, displayed a distinct endothelial profile of activity comprising detrimental effects on mitochondria and cellular viability and suppression of endothelial ET-1 release possibly interfering with ET-1–dependent local regulation of endothelial permeability.

## 1. Introduction

Nrf2—nuclear factor (erythroid 2-related factor) 2—is the primary player in the inducible cell defence system that regulates expression of over 600 target genes including detoxification, cytoprotective, and antioxidant enzymes; ABC transporters; and other stress response enzymes and proteins [1, 2]. The plethora of products encoded by these genes includes antioxidant and cytoprotective genes (e.g. genes related to glutathione synthesis, glutathione S-transferases, thioredoxin, peroxiredoxins, hemoxygenase-1, and ferritin) and many others, i.e., regulators of transcription, growth factors, and proteins responsible for xenobiotic metabolism and clearance (reviewed in Baird and Dinkova-Kostova [3]).

Given the important role of Nrf2 in cellular defence, it was not a surprise that Nrf2-/- mice were more susceptible to oxidative stress-induced diseases, including acute lung injury, chronic obstructive pulmonary diseases, diabetic nephropathy, heart failure, and cancer [4]. On the other hand, Nrf2 pathway activation exerts a wide range of protective functions. There is a substantial interest in identifying and developing Nrf2 activators that could be exploited therapeutically in conditions related to oxidative stress and inflammation, such as multiple sclerosis and complications of diabetes (e.g., retinopathy and nephropathy), as well as many other diseases including solid tumors, lymphomas, and neurodegenerative diseases [1, 3–9].

L-sulforaphane, an isothiocyanate present in cruciferous vegetables (broccoli), was one of the first studied activators of Nrf2. This compound was tested in a phase 2 clinical study in men with recurrent prostate cancer [10], but the expected therapeutic effects were not achieved. However, in type 2 diabetic patients, L-sulforaphane-rich broccoli sprout powder caused a significant improvement in serum insulin concentration, glucose-to-insulin ratio, and insulin resistance [11].

Another Nrf2 inducer, dimethyl fumarate, is a methyl ester of fumaric acid—a metabolite of the citric acid cycle in mitochondria. Dimethyl fumarate was tested in the treatment of psoriasis where it decreased the number of T-cells via the activation of apoptosis [12] and has been used in the treatment of psoriasis for many years [13]. Importantly, this compound has been shown to be efficacious in the treatment of multiple sclerosis [14]. In a phase 3 clinical study in relapsing-remitting multiple sclerosis patients, dimethyl fumarate was shown to reduce the progression of disability [15], and the compound was approved by FDA in 2013 for the treatment of relapsing-remitting multiple sclerosis [16].

Some of the most promising Nrf2 inducers are the derivatives of oleanolic acid [17]. A semisynthetic triterpenoid derivative named bardoxolone methyl (also known as CDDO-Me) is a potent Nrf2 inducer that stimulates Nrf2-dependent cytoprotective responses in nanomolar concentrations [13, 17]. In multiple preclinical studies, bardoxolone methyl was shown to reduce diabetic complications, cancer, cardiovascular disease, neurodegenerative diseases, and chronic obstructive pulmonary disorder [13, 17] and numerous clinical trials were launched [1, 13, 17]. However, in October 2012, a phase 3 BEACON trial was terminated. This clinical study (Bardoxolone Methyl Evaluation in Patients with Chronic Kidney Disease and Type 2 Diabetes Mellitus: The occurrence of renal events (BEACON)) was a phase 3 randomized, double-blind, placebo-controlled trial designed to determine whether bardoxolone methyl could reduce End-Stage Renal Disease (ESRD) and cardiovascular events [18]. The study was terminated due to safety concerns and a significantly increased risk of heart failure requiring hospitalization, which also increased the composite cardiovascular outcome (nonfatal myocardial infarction, nonfatal stroke, hospitalization for heart failure, or death from cardiovascular causes) in the bardoxolone methyl-treated group compared to placebo [18]. Increased incidence of cardiovascular events in bardoxolone methyl-treated patients in the BEACON trial was attributed to the ability of bardoxolone methyl to modulate the endothelin pathway [18, 19]. An excess rate of heart failure hospitalization among those assigned to the bardoxolone-treated group was linked to fluid overload in patients at risk [20] and seemed to recapitulate those observed with endothelin receptor antagonists in patients with advanced CKD [19, 21]. Furthermore, preclinical studies demonstrated the suppressive effects of bardoxolone methyl on endothelin signalling in the kidneys by reducing the expression of the ET_A_ receptor protein, independently of bardoxolone methyl-induced improvement of eGFR and preservation of kidney function [19]. Given the fact that ET-1 plays an important role in the regulation of sodium and water homeostasis [22], it was claimed that bardoxolone methyl may pharmacologically promote acute retention of sodium and water through the modulation of the endothelin pathway [21]. However, this hypothesis could not fully explain the symptoms observed in patients suffering from bardoxolone methyl-induced side effects [23], particularly when post hoc analysis confirmed improved kidney function and eGFR in bardoxolone methyl-treated patients [21]. Furthermore, ET antagonists have different hemodynamic effects from those induced by bardoxolone methyl, and generally, suppression of the endothelin pathway should rather be protective than detrimental against an adverse cardiovascular event [24–26]. Moreover, the manner in which bardoxolone methyl modulates the endothelin pathway in other tissues including the endothelium has yet to be examined, and it is not known whether bardoxolone methyl displays any detrimental effect on endothelial function—in particular the endothelial barrier—that could possibly result in peripheral oedema. Endothelial function determines heart failure progression not only by an NO-dependent action that is known to have prognostic significance in heart failure patients [27] but also by regulating the barrier function in microcirculation. Indeed, changes in endothelial permeability and vascular leakage could contribute to drug-induced progression of heart failure and peripheral oedema, and such detrimental mechanism in diabetic patients was reported for rosiglitazone [28], a peroxisome proliferator-activated receptor-*γ* (PPAR-*γ*) agonist, shown to significantly increase the risk of heart failure [29]. Therefore, we aimed to test the hypothesis that the detrimental effects of bardoxolone methyl in the BEACON trial could be, at least partially, explained by bardoxolone-induced effects on endothelial function. For that purpose, in the present work we characterize the effects of bardoxolone methyl on microvascular human endothelium toxicity, mitochondrial function, endothelin-1 release, and endothelial permeability. The effects of bardoxolone methyl were compared with those of two other well-characterized Nrf2 activators used in clinical studies: dimethyl fumarate and L-sulforaphane.

## 2. Methods

### 2.1. Cell Culture and Drug Treatment

Human dermal microvascular endothelial cells, HMEC-1 (purchased from ATCC, Cat. No. CRL3243™), were cultured in MCDB 131 Medium (Thermo Fisher Scientific) supplemented with FBS (10%, Thermo Fisher Scientific), L-glutamine (2 mM, Thermo Fisher Scientific), hydrocortisone (0.05 mg/ml, Sigma-Aldrich), Epidermal Growth Factor (5 ng/ml), 100 U/ml penicillin, 10 *μ*g/ml streptomycin, and 250 ng/ml amphotericin B (Sigma-Aldrich). HMEC-1 were cultured under standard conditions (37°C, 5% CO_2_) and passaged two times a week. In all experiments, cells between the second and tenth passages were used only when they reached full postplating confluency. Bardoxolone methyl (CDDO methyl ester, Cayman Chemical), dimethyl fumarate (Sigma-Aldrich), and L-sulforaphane (Sigma-Aldrich) initially diluted in DMSO were added to the culture medium at final concentrations of 100 nM, 300 nM, 500 nM, 1 *μ*M, 3 *μ*M, and 5 *μ*M in triplicates, if not stated otherwise. Control cells were treated with 0.05% DMSO added to the culture medium. To check for acute toxicity, the cells were incubated with tested agents for 3 hours. Standard toxicity assessment time was set to 24 hours, according to the literature [30].

### 2.2. Assessment of Nrf2 Nuclear Expression

HMECs were seeded at a density of 1,000,000 per well and grown in 6-well plates until they reached full confluence, then cells were incubated with bardoxolone methyl, dimethyl fumarate, and L-sulforaphane for three hours. To measure Nrf2 expression in the nucleus, a Nuclear Extract Kit (Active Motif) was used to isolate the nuclear and cytoplasmic fractions. Nuclear and cytoplasmic extracts (25 or 30 *μ*g protein/sample) were processed as follows: first, the membranes were scanned for total protein content for further band normalization, and subsequently, they were incubated with primary rabbit polyclonal Anti-Nrf2 Antibody (H-300) (sc-13032, Santa Cruz Biotechnology, lot No. GR197455-1) 1 : 1000 overnight and with goat anti-rabbit secondary antibody (sc-2004, Santa Cruz Biotechnology, lot No. 12314) 1 : 2500 for 45 minutes. After chemiluminescent band detection, membranes were washed and incubated with primary Anti-Lamin A/C Monoclonal Antibody (mab636, Thermo Fisher Scientific, lot No. QF215120) 1 : 1000 overnight and with anti-mouse secondary antibody (sc-516102, Santa Cruz Biotechnology) 1 : 5000 for 1 hour. Bands were again detected with the use of chemiluminescence, measured and normalized to total protein content using Image Lab Software (Bio-Rad).

### 2.3. Assessment of ROS Production

HMECs were cultured in 96-well plates (seeding density: 15,000 cells per well) and treated with bardoxolone methyl, dimethyl fumarate, and L-sulforaphane for 24 hours and incubated for 15 minutes with the following fluorescent dyes: dihydroethidium DHE (2 *μ*g/ml, Thermo Fisher Scientific) and Hoechst 33342 (0.5 *μ*l/ml, Thermo Fisher Scientific) at 37°C. After washing, images were captured with an Olympus Scan^R automated fluorescence microscope (Olympus Corporation) with the use of 20x magnification in two channels: DAPI for nuclei localization (Hoechst 33342, ex/em 346/460 nm) and Texas Red for reactive oxygen species/reactive nitrogen species (ROS/RNS) indication (DHE, ex/em 518/606 nm). Image analysis was performed with the use of a Columbus Image Data Storage and Analysis System (Perkin Elmer), and mean fluorescence intensity was normalized to the number of living cells.

### 2.4. Assessment of Endothelial Toxicity

#### 2.4.1. MTS Tetrazolium Assay

HMECs were plated into 96-well plates at a density of 15,000 per well in order to produce confluency on the second postplating day. The cells were incubated with bardoxolone methyl, dimethyl fumarate, and L-sulforaphane for 3 and 24 hours. Cell viability was determined with use of a Non-Radioactive Cell Proliferation Assay (Promega) according to the manufacturer's instructions. The absorbance was recorded at 490 nm using a Synergy4 plate reader (BioTek). Cells were then washed with PBS and suspended in Mammalian Protein Extraction Reagent (Thermo Fisher Scientific) containing phosphatase (PhosSTOP, Roche) and protease (cOmplete, Roche) inhibitors and frozen at -20°C. The next day, the samples were thawed and total protein concentration was determined with use of a colorimetric BCA Protein Assay Kit (Thermo Fisher Scientific). Absorbance was recorded at 562 nm. The results of the MTS test were normalized to the protein concentration of the sample and calculated as a percent of control cells.

#### 2.4.2. Annexin V/Propidium Iodide Flow Cytometry Assay

Flow cytometry was used to determine cellular apoptosis and necrosis. HMECs were cultured in 24-well plates (500,000 per well) and incubated for 3 and 24 hours with bardoxolone methyl, dimethyl fumarate, and L-sulforaphane (single wells). After incubation, staining with Annexin V and propidium iodide was performed with the use of a FITC Annexin V Apoptosis Detection Kit (BD Biosciences) according to the manual. The cells were analyzed with an LSR II flow cytometer (Becton Dickinson).

#### 2.4.3. Cell Counting

HMECs were cultured as described above (Section 2.4.1) and treated with bardoxolone methyl (100 nM-10 *μ*M), dimethyl fumarate (500 nM-1 mM), and L-sulforaphane (500 nM-100 *μ*M) for 24 hours. Afterwards, the cells were washed with PBS 1x, fixed with 4% formaldehyde for 10 min at RT, and incubated for 30 minutes at 37°C with the following fluorescent dyes: Hoechst 33258 (1 *μ*g/ml, Sigma-Aldrich) for nucleic acid staining and YO-PRO-1 Iodide (1 : 1000, Thermo Fisher Scientific) for identification of apoptotic cells. Cell imaging was performed with a Confocal Quantitative Image Cytometer CQ1 (Yokogawa Electric Corporation) with the use of 10x magnification at two wavelengths: 405/452 nm (blue) and 488/525 nm (green). Image analysis was performed using a Columbus Image Data Storage and Analysis System (Perkin Elmer).

### 2.5. Assessment of Mitochondrial Function

Cellular bioenergetics were measured with use of the Seahorse XFe96 Analyzer (Agilent Technologies). Forty-eight hours prior to the start of the experiment, cells were seeded at a density of 19,500 per well into 96-well XF cell culture plates, according to the manufacturer's protocol. Before the start of experiments, cells were washed twice with 200 *μ*l of bicarbonate-free low-buffered assay medium (containing 10 mM glucose, 1 mM pyruvate, and 2 mM glutamine, pH = 7.4) and preincubated for 1 hour with bardoxolone methyl, dimethyl fumarate, and L-sulforaphane diluted in assay medium. Changes in cellular respiration were assessed for 2 hours (total time of incubation with Nrf2 activators was three hours). During the assay, sequential injections of 1 *μ*g/ml oligomycin (an inhibitor of mitochondrial ATPase/ATP synthetase), 0.7 *μ*M FCCP (trifluoromethoxy carbonylcyanide phenylhydrazone—a protonophore and uncoupler of oxidative phosphorylation), and 1 *μ*M rotenone/antimycin A (an inhibitor of mitochondrial electron transport at NADH : ubiquinone oxidoreductase and an inhibitor of electron transfer at complex III) were performed. Results regarding the following parameters—basal respiration, proton leak, maximal respiration, spare respiratory capacity, nonmitochondrial respiration, and ATP production—were obtained with use of a test report generator provided by Agilent Technologies.

Mitochondrial membrane potential was evaluated using JC-1, which is a cationic dye that accumulates inside mitochondria. Depending on the value of the mitochondrial membrane potential, it forms either red fluorescent aggregates or green fluorescent monomers. A decrease in the red/green fluorescence intensity ratio indicates mitochondrial depolarization. HMECs were plated into 96-well plates at a density of 40,000 per well in order to produce confluency on the first postplating day. After 3 hours of incubation with bardoxolone methyl, dimethyl fumarate, and L-sulforaphane, cells were washed with PBS and incubated for 30 minutes at 37°C in the dark with 1 *μ*g/ml JC-1 (Thermo Fisher Scientific). Shortly before JC-1 addition, 100 *μ*M FCCP was added to the cells incubated with culture media to serve as a positive control. Cell imaging was performed with a Confocal Quantitative Image Cytometer CQ1 (Yokogawa Electric Corporation) with the use of 20x magnification at two wavelengths: 561/617 nm (red) and 488/525 nm (green). Image analysis was performed using a Columbus Image Data Storage and Analysis System (Perkin Elmer).

### 2.6. Assessment of Endothelin-1 Release

HMECs were plated into 96-well plates at a density of 15,000 per well and treated with bardoxolone methyl, dimethyl fumarate, and L-sulforaphane for 3 and 24 hours in duplicates. After the assigned time, cell culture supernatants were collected, centrifuged, and stored at -80°C. Endothelin-1 was quantified with use of an ELISA Kit (R&D Systems). Total protein concentration was determined as described above and the results were normalized.

### 2.7. Assessment of Endothelial Permeability

To measure changes in endothelial permeability, we used the ECIS methodology based on the measurement of cellular electric potential [31, 32]. HMEC-1 cells were seeded at a density of 30,000 per well into special 96-well plates containing golden electrodes (Applied Biophysics). An ECIS array holder was placed into the incubator, and cells were grown for approximately ten days until they reached full confluence confirmed by ECIS readout (capacitance values: 0.5–1.0 nanoFarads). One day prior to the start of the experiment, fully supplemented culture medium was changed to medium without FBS. Endothelin-1 (100, 300, 500, and 1000 pM), selective antagonists of ET_A_ and ET_B_ receptors BQ123 and BQ788 (1 *μ*M), bardoxolone methyl, dimethyl fumarate, and L-sulforaphane (0.1–5 *μ*M) were added into the wells in quadruplicate and the impedance was measured throughout 24 hours. Control wells were treated with fresh culture medium (no FBS), 0.05% DMSO, 5 *μ*M forskolin, and 10 *μ*M histamine.

### 2.8. Statistical Analysis

All parameters were expressed as mean ± standard error of the mean (SEM). The data from each experiment (repeated three times or more) were first processed with the Shapiro-Wilk normality test, and all data were normally distributed. Intergroup differences were assessed by one-way analysis of variance (ANOVA), followed by an adequate post hoc test (Duncan's, Dunnett's, or Dunn's), if appropriate.

## 3. Results

### 3.1. Effects of Bardoxolone Methyl, Dimethyl Fumarate, and L-Sulforaphane on Nrf2 Expression in HMECs

**Figure 1:**
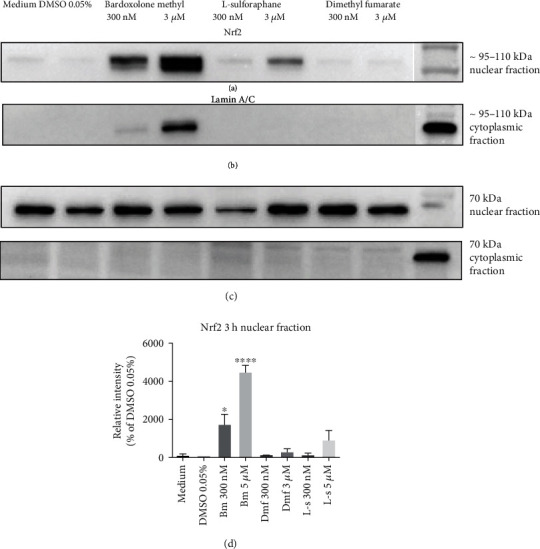
Effects of Nrf2 activators on Nrf2 expression in endothelial cells. A representative Western blot analysis of Nrf2 expression in nuclear (a, d) and cytoplasmic (b) fraction lysates obtained from HMEC-1 cells treated for 3 hours with 300 nM and 3 *μ*M of bardoxolone methyl (Bm), dimethyl fumarate (Dmf), L-sulforaphane (L-s), culture medium alone, or with the addition of 0.05% DMSO. The expression of Lamin A/C was also determined to define fraction purity and serve as loading control (c). The results (d) are presented as a % of control (DMSO 0.05%) ± SEM, *n* = 3. The significance of the differences between the means was evaluated by one-way analysis of variance (ANOVA) with Dunnett's post hoc test; ^∗^*p* < 0.05 and ^∗∗∗∗^*p* < 0.0001 indicate significant difference vs. the control group (DMSO 0.05%). The results were obtained in three independent experiments. In all panels, the first lane on the right contains a protein molecular weight marker.

To compare the effects of bardoxolone methyl, dimethyl fumarate, and L-sulforaphane on Nrf2 nuclear expression in HMECs, the expression of Nrf2 in the nuclear fractions was assessed by means of Western blots. As shown in Figure 1, bardoxolone methyl at concentrations of 300 nM and 3 *μ*M increased Nrf2 expression in the nuclear fraction of HMECs by 17.5-fold and 45-fold, respectively. L-sulforaphane appeared to be less potent and dimethyl fumarate the least potent activator of Nrf2 because at a concentration of 3 *μ*M, these compounds increased Nrf2 expression in the nuclear fraction of HMECs by 9.3- and 3.1-fold, respectively (Figure 1(a)). In the cytoplasmic fraction, a pronounced increase in the level of Nrf2 was present only after treatment with 3 *μ*M of bardoxolone methyl (Figure 1(b)). Expression of Lamin A/C was detected in the nuclear extracts, while it was absent in the cellular fractions, confirming the nuclear fraction purity (Figure 1(c)).

### 3.2. Effects of Bardoxolone Methyl, Dimethyl Fumarate, and L-Sulforaphane on Reactive Oxygen Species (ROS) Generation in HMECs

**Figure 2:**
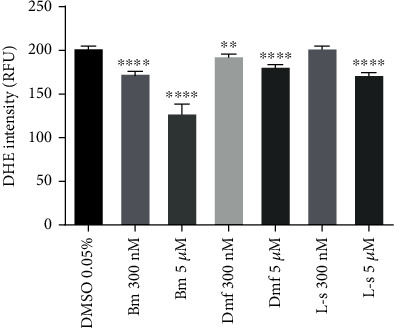
Effects of Nrf2 activators on reactive oxygen species production in endothelial cells. HMEC-1 cells were incubated for 24 hours with bardoxolone methyl (Bm), dimethyl fumarate (Dmf), and L-sulforaphane (L-s), (300 nM and 5 *μ*M). Intensity values of DHE fluorescence are expressed as relative fluorescence units ± SEM, *n* = 3. The significance of the differences between the means was evaluated by one-way analysis of variance (ANOVA) with Dunnett's post hoc test; ^∗∗^*p* < 0.01 and ^∗∗∗∗^*p* < 0.0001 indicate significant difference vs. the control group (DMSO 0.05%). The results were obtained in three independent experiments.

Incubation of HMECs with bardoxolone methyl for twenty four hours at a concentration of 300 nM resulted in a significant decrease in ROS production measured by DHE fluorescence, and this effect was even more pronounced in the cells treated with 5 *μ*M bardoxolone methyl. Under similar experimental conditions, dimethyl fumarate also produced a decrease in the DHE signal, but this effect was clearly visible at the concentration of 5 *μ*M. Similarly, 5 *μ*M of L-sulforaphane significantly lowered the DHE signal in HMECs (Figure 2).

### 3.3. Endothelial Toxicity of Bardoxolone Methyl, Dimethyl Fumarate, and L-Sulforaphane in HMECs

**Figure 3:**
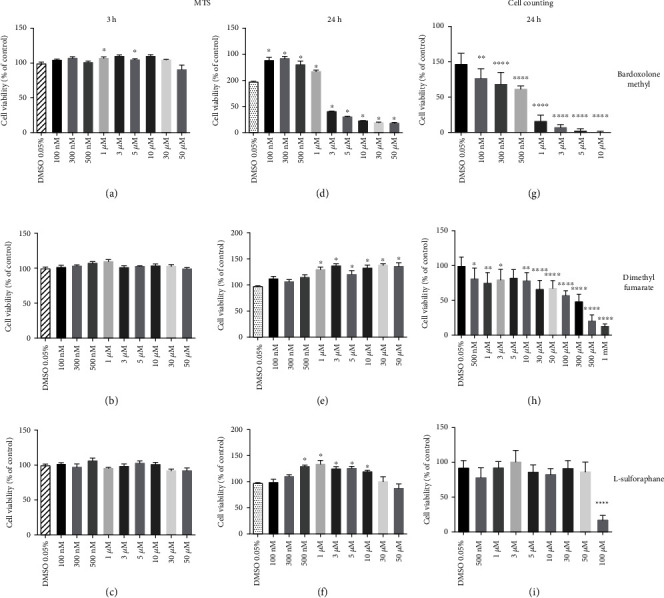
Effects of Nrf2 activators on endothelial viability assessed by the MTS test and cell counting. HMEC-1 cells were incubated with bardoxolone methyl, dimethyl fumarate, and L-sulforaphane (100 nM-50 *μ*M) for 3 (a–c) and 24 (d–f) hours for the MTS test. Concentrations ranging from 100 nM up to 1 mM were applied in the cell counting test (g–i). Results are expressed as a % of control (cells treated with 0.05% DMSO). Values are expressed as mean ± SEM, *n* = 4. The significance of the differences between the means was evaluated by one-way analysis of variance (ANOVA) with Duncan's (MTS) or Dunnett's (cell counting) post hoc test if appropriate; ^∗^*p* < 0.05, ^∗∗^*p* < 0.01, and ^∗∗∗∗^*p* < 0.0001 indicate significant difference vs. the control group (DMSO 0.05%). The results were obtained in four independent experiments.

As shown in Figure 3, bardoxolone methyl, dimethyl fumarate, and L-sulforaphane after a 3-hour-long incubation did not cause any significant changes in cell viability assessed by the MTS reduction assay, with the exception of 1 and 5 *μ*M bardoxolone methyl (Figures 3(a)–3(c)). However, after a 24-hour-long treatment, bardoxolone methyl at a concentration range of 100 nM–1 *μ*M caused an increase in normalized MTS reduction, while higher concentrations caused a substantial decrease in the parameter (IC_50_ = 3.23 *μ*M) (Figure 3(d)). In the same experimental conditions (24-hour-long drug treatment), dimethyl fumarate given at micromolar concentrations (1–50 *μ*M) caused an increase in MTS reduction (Figure 3(e)). L-sulforaphane increased MTS reduction at the concentration range of 500 nM–10 *μ*M but not at the highest concentrations used (30–50 *μ*M) (Figure 3(f)). To confirm the obtained results, the effects of Nrf2 activators on the cell count was tested using an extended concentration range. Bardoxolone methyl did cause a significant decrease in the number of living cells in the tested concentration range after 24 hours of treatment (Figure 3(g)). Cell counting revealed increasing dose-dependent toxicity of dimethyl fumarate (IC_50_ = 95.52 *μ*M) at the concentration range of 500 nM–1 mM (Figure 3(h)). Due to the compound solubility, the highest concentration of L-sulforaphane that could be tested in order to not exceed a 0.05% concentration of DMSO was 100 *μ*M, at the same time being the only toxic concentration. The cells treated with 500 nM–50 *μ*M did not show neither an increase nor a decrease in cellular viability (Figure 3(i)).

To characterize further effects of Nrf2 activators on cellular viability, apoptotic and necrotic cells were quantified by flow cytometry with use of Annexin V and propidium iodide. As shown in Table 1, HMECs treated for 24 hours with bardoxolone methyl (3 and 5 *μ*M) but not with dimethyl fumarate or L-sulforaphane displayed a significantly diminished subpopulation of live cells and increased subpopulations of necrotic and apoptotic+necrotic cells. There was no significant difference between the control and experimental groups in the composition of the cell population regarding live, apoptotic, necrotic, and apoptotic+necrotic cells.

### 3.4. Effects of Bardoxolone Methyl, Dimethyl Fumarate, and L-Sulforaphane on Mitochondrial Respiration and Mitochondrial Membrane Potential in HMECs

**Figure 4:**
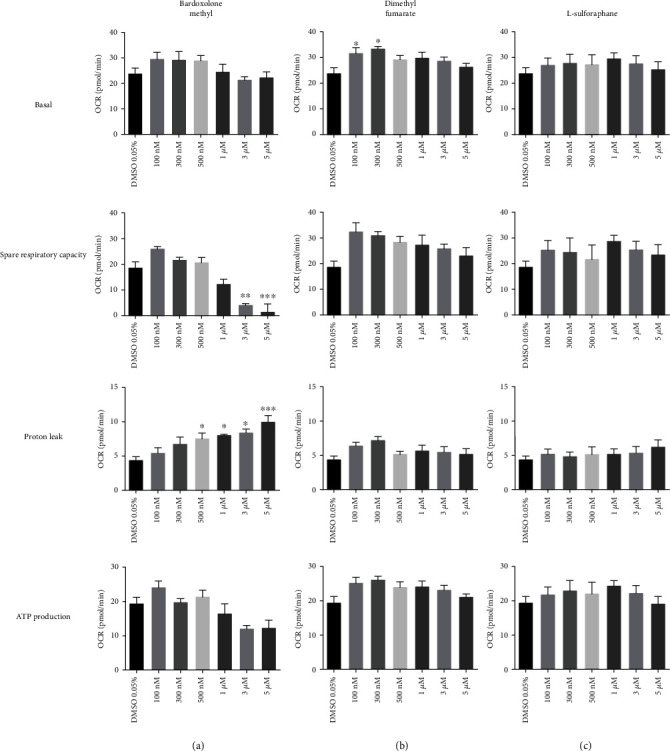
Effects of Nrf2 activators on mitochondrial function in endothelial cells. HMEC-1 cells were incubated for three hours with bardoxolone methyl (a), dimethyl fumarate (b), and L-sulforaphane (c) (100 nM–5 *μ*M). Values are expressed as mean oxygen consumption rate (OCR, in pmol/min ± SEM, *n* = 5). The significance of the differences between the means was evaluated by one-way analysis of variance (ANOVA) with Dunnett's post hoc test if appropriate; ^∗^*p* < 0.05, ^∗∗^*p* < 0.01, and ^∗∗∗^*p* < 0.001 indicate significant difference vs. the control group (DMSO 0.05%). The results were obtained in five independent experiments.

To characterize the effects of Nrf2 activators on endothelial bioenergetics, a mitochondrial stress assay was performed in HMECs treated with bardoxolone methyl, dimethyl fumarate, or L-sulforaphane for 3 hours. As shown in Figure 4, among all three Nrf2 activators, bardoxolone methyl exerted the most pronounced actions: it significantly increased proton leakage in a concentration-dependent manner (0.5–5 *μ*M), decreased spare respiratory capacity (3–5 *μ*M), and showed a tendency to decrease ATP production that, however, did not reach statistical significance (Figure 4(a)). Although low concentrations (100 and 300 nM) of dimethyl fumarate increased basal respiration and showed a tendency to increase spare respiratory capacity and ATP production (Figure 4(b)), there were no other significant effects of dimethyl fumarate on HMEC bioenergetics. L-sulforaphane did not change any of the bioenergetic parameters in a statistically significant fashion (Figure 4(c)).

**Figure 5:**
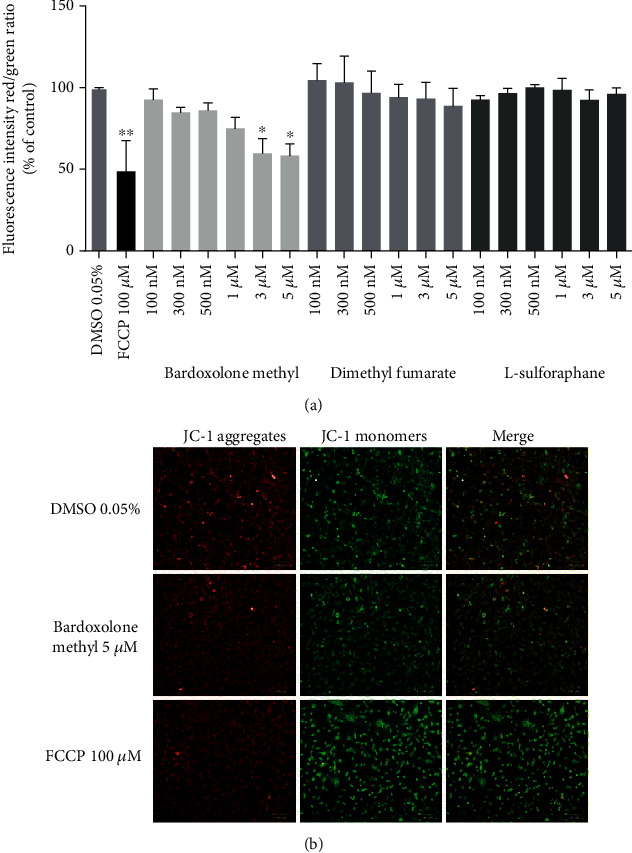
Effects of Nrf2 activators on mitochondrial membrane potential in endothelial cells. HMEC-1 cells were incubated for three hours with FCCP (100 *μ*M) or with bardoxolone methyl, dimethyl fumarate, and L-sulforaphane (100 nM–5 *μ*M) (a). Values are expressed as a ratio of red and green fluorescence intensity of JC-1 calculated as a % of control ± SEM, *n* = 3. The significance of the differences between the means was evaluated by one-way analysis of variance (ANOVA) with Duncan's post hoc test if appropriate; ^∗^*p* < 0.05 and ^∗∗^*p* < 0.01 indicate significant difference vs. the control group (DMSO 0.05%). (b) Representative images of HMEC-1 cells treated with 0.05% DMSO (upper panel), 5 *μ*M of bardoxolone methyl (middle panel), and 100 *μ*M of FCCP (lower panel). Images were collected with the use of 20x magnification at 561/617 nm (red fluorescence, JC-1 aggregates), 488/525 nm (green fluorescence, JC-1 monomers), and merged. The results were obtained in three independent experiments.

Detrimental effects of bardoxolone methyl on mitochondria in endothelial cells were confirmed by the demonstration that bardoxolone methyl (3 and 5 *μ*M) decreased mitochondrial membrane potential as evidenced by a decrease in the red/green fluorescence intensity of the JC-1 aggregate/monomer ratio, while dimethyl fumarate and L-sulforaphane did not have any effect (Figure 5).

### 3.5. Effects of Bardoxolone Methyl, Dimethyl Fumarate, and L-Sulforaphane on Endothelin-1 Release in HMEC Culture

**Figure 6:**
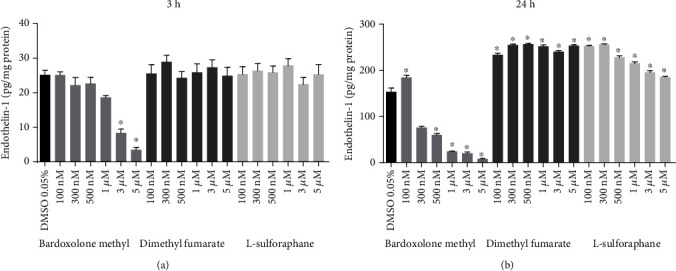
Effects of Nrf2 activators on secretion of endothelin-1 from endothelium to culture media. HMEC-1 cells were incubated for 3 (a) and 24 (b) hours with bardoxolone methyl, dimethyl fumarate, and L-sulforaphane (100 nM–5 *μ*M). Values are expressed as mean ± SEM, *n* = 3. The significance of the differences between the means was evaluated by one-way analysis of variance (ANOVA) with Dunnett's post hoc test if appropriate; ^∗^*p* < 0.0001 indicates significant difference vs. the control group (DMSO 0.05%). The results were obtained in three independent experiments.

Bardoxolone methyl, but not the other Nrf2 activators, caused a decrease in ET-1 concentration in the medium, even upon a short-term, 3-hour-long incubation of HMECs with the compound (Figure 6(a)). The effect of bardoxolone methyl on ET-1 release was more pronounced (and concentration dependent) when the incubation period was prolonged up to 24 hours (Figure 6(b)). Dimethyl fumarate at almost all tested concentrations (0.1–5 *μ*M) slightly increased ET-1 production in HMECs. L-sulforaphane in the lower concentration range (0.1–0.3 *μ*M) slightly increased ET-1 production in HMECs, while a higher concentration of L-sulforaphane (3–5 *μ*M) decreased ET-1 release in HMECs. The inhibitory effect of L-sulforaphane on ET-1 release, however, was much less pronounced compared with that of bardoxolone methyl (Figure 6(b)) and was not observed after a 3-hour-long incubation (Figure 6(a)).

### 3.6. Effects of Bardoxolone Methyl, Dimethyl Fumarate, and L-Sulforaphane on Endothelial Permeability in HMECs

**Figure 7:**
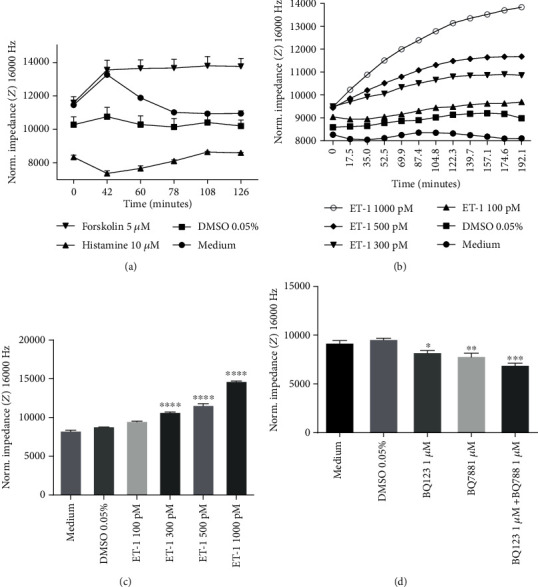
Effects of endothelin-1 and endothelin-1 receptor antagonists on endothelial permeability. The measurement of impedance was performed using an ECIS assay at a frequency of 16,000 Hz in HMEC-1 cells: (a) incubated for one hour with medium, DMSO 0.05%, histamine 10 *μ*M, and forskolin 5 *μ*M; (b and c) incubated for three hours with endothelin-1 (ET-1) (100 pM–1000 pM); (d) incubated for three hours with 1 *μ*M of BQ123 and/or BQ788. Values are expressed as mean normalized impedance ± SEM, *n* = 3. The significance of the differences between the means was evaluated by one-way analysis of variance (ANOVA) with Dunnett's post hoc test if appropriate; ^∗^*p* < 0.05, ^∗∗^*p* < 0.01, ^∗∗∗^*p* < 0.001, and ^∗∗∗∗^*p* < 0.0001 indicate significant difference vs. the control groups: medium (a) and DMSO 0.05% (b, c, and d). The results were obtained in three independent experiments.

As shown in Figure 7, histamine (10 *μ*M) decreased and forskolin (5 *μ*M) increased impedance, showing an increase and a decrease in endothelial layer permeability, respectively (Figure 7(a)), thus supporting the reliability of our experimental setup to study the endothelial barrier. To link the effects of Nrf2 activators on endothelial permeability in HMECs with ET-1 function, the effect of ET-1 given alone on endothelial permeability was assessed. ET-1 caused a concentration-dependent decrease in permeability (Figures 7(b) and 7(c)), while ET-1 receptor antagonists BQ123 and BQ 788 increased endothelial permeability (Figure 7(d)), as evidenced by increased and decreased impedance, respectively.

**Figure 8:**
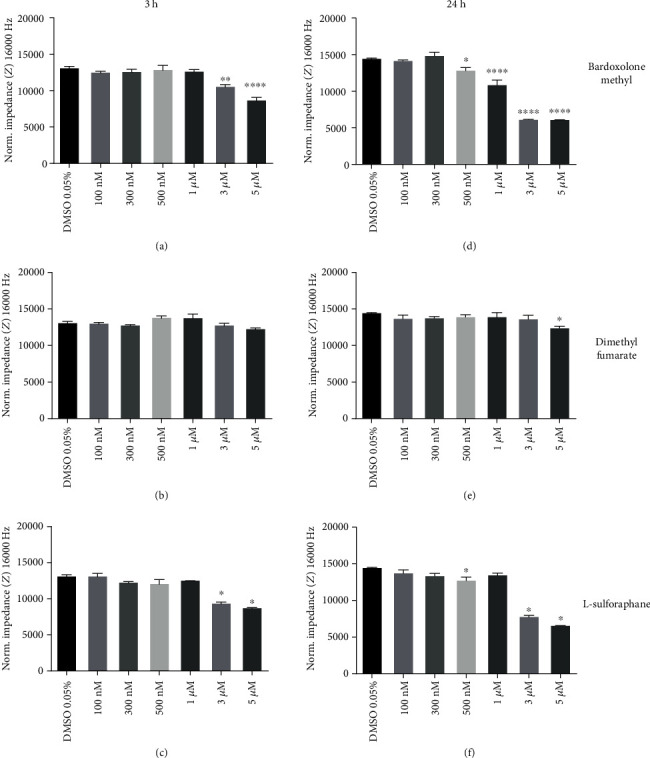
Effects of Nrf2 activators on endothelial permeability. The measurement of impedance was performed using an ECIS assay at a frequency of 16,000 Hz in HMEC-1 cells incubated for 3 and 24 hours with bardoxolone methyl (a and d), dimethyl fumarate (b and e), or L-sulforaphane (100 nM–5 *μ*M) (c and f). Values are expressed as mean normalized impedance ± SEM, *n* = 3. The significance of the differences between the means was evaluated by one-way analysis of variance (ANOVA) with Duncan's post hoc test if appropriate; ^∗^*p* < 0.05, ^∗∗^*p* < 0.01, and ^∗∗∗∗^*p* < 0.0001 indicate significant difference vs. the control group (DMSO 0.05%). The results were obtained in three independent experiments.

Bardoxolone methyl (3 and 5 *μ*M), even after 3 hours of incubation (that did not cause endothelial toxicity (Figure 3, Table 1) but lowered ET-1 production (Figure 6)), significantly decreased the impedance indicating an increase in the permeability of the endothelial monolayer (Figure 8(a)), while dimethyl fumarate did not disturb endothelial barrier function and L-sulforaphane at the highest concentrations had only minor effects after 3 hours of incubation (Figures 8(b) and 8(c)). After a 24-hour-long period of incubation, a further increase in permeability of endothelial cells was observed in the cells treated with micromolar concentrations of bardoxolone methyl, an effect shared also by L-sulforaphane (Figures 8(d) and 8(f)). In these experimental conditions (24 hour incubation), dimethyl fumarate did not affect endothelial permeability (Figure 8(e)).

## 4. Discussion

To the best of our knowledge, in the present work, we have demonstrated for the first time that bardoxolone methyl affects mitochondrial function, cellular viability, ET-1 release, and endothelial barrier function in human microvascular endothelium, and those effects are not shared with dimethyl fumarate and L-sulforaphane tested in the same concentration range.

Clearly, the beneficial effect of Nrf2 on vascular endothelial function has been repeatedly reported and evidenced, also in our study by, e.g., a decrease in ROS production. Such effects include not only a decrease in vascular oxidative stress but also a downregulation of endothelial proinflammatory adhesion molecule expression [33] and restoration of endothelial function in hypertension, atherosclerosis, diabetes, or aging [7, 34, 35]. Here, by means of flow cytometry, we detected direct endothelial toxicity for bardoxolone methyl (at concentrations 3–5 *μ*M), but not for other Nrf2 activators, which was not equivocally supported by the MTS assay in any of the experimental groups, as the latter assay may show divergent results if mitochondrial activity is altered. Indeed, bardoxolone methyl seems to have a biphasic effect: it sustains cellular metabolism at lower doses and becomes cytotoxic in higher concentrations; similarly, dimethyl fumarate increased MTS readouts.

To better understand the mechanisms involved in bardoxolone methyl-induced endothelial toxicity, we analyzed the effects of bardoxolone methyl compared with other Nrf2 activators on cellular bioenergetics and mitochondrial membrane potential. Although the main source of energy in the endothelium is glycolysis [36], mitochondrial activity and redox signalling plays an important role in maintaining endothelial integrity [37]. We showed concentration-dependent effects of bardoxolone methyl on mitochondrial activity comprising proton leak, spare respiratory capacity, and mitochondrial membrane potential. Interestingly, in cells treated with bardoxolone methyl, an increase in proton leak was observed even at the nontoxic concentration of 500 nM. The uncoupling effect could be beneficial for the cell [38–40]; however, the uncoupling effect of bardoxolone methyl was severe and contributed to endothelial toxicity, in particular in the presence of higher concentrations of bardoxolone methyl. Other Nrf2 activators—dimethyl fumarate and L-sulforaphane—displayed neither endothelial toxicity nor detrimental effects on mitochondrial bioenergetics or mitochondrial membrane potential, even at the highest concentrations used. The endothelial toxicity of bardoxolone methyl was supposedly an Nrf-2-independent effect, since increased Nrf2 activity may modulate mitochondrial function and has a protective, rather than detrimental effect on mitochondrial integrity [41, 42]. On the other hand, the effects of dimethyl fumarate on respiration could be linked to the metabolism of dimethyl fumarate to fumarate, feeding the citric acid cycle [16].

Many Nrf2-activating chemicals including bardoxolone methyl are electrophilic, and their mechanisms of action are based on the modification of cysteine residues in Keap1, resulting in an impairment of Keap1 function, inhibition of the ubiquitin E3 ligase activity of the Keap1-Cul3 complex, and subsequently leading to Nrf2 activation. Electrophiles are able to target distinct cysteine residues as well as lead to rapid and selective depletion of mitochondrial glutathione [43] implicating, at least partially, unspecific mechanisms of action of these types of compounds [44]. Proteomic analysis revealed that bardoxolone methyl interacts with 577 cellular proteins [45]. It was suggested that the side effects of bardoxolone methyl may be attributed to its highly reactive *α*-cyano-*α*, *β*-unsaturated ketone (CUK) moiety in ring A, which avidly reacts with other proteins besides Keap1. In fact, modification of the CUK moiety in ring A results in a marked decrease in cytotoxicity [45]. Thus, it may well be that the mitochondrial and cellular toxicity of bardoxolone methyl reported here was due to an unspecific modification of a thiol-containing mitochondrial protein, representing a known mechanism of cellular toxicity described for other agents [46]. Noteworthy, other mechanisms of bardoxolone methyl-induced cellular toxicity reported for cancer cells could also be involved in inducing endothelial toxicity by this compound [47]. Interestingly, it was demonstrated that dimethyl fumarate reduced cellular maximal respiratory and reserve capacity and these effects were completely inhibited by N-acetyl cysteine, again suggesting the involvement of thiols [48]. These experiments were conducted in retinal epithelial cells at a concentration of 10 *μ*M and a longer incubation period compared to our study. Also, L-sulforaphane was shown to inhibit the proliferation of endothelial cells, although the concentrations used were higher than those investigated in our study [49]. These reports indicate that unspecific detrimental effects of Nrf2 activators on mitochondrial bioenergetics and cellular function may be seen not only with bardoxolone but also with other electrophilic activators of Nrf2 in higher concentrations and in a tissue-specific manner.

Increased risk of heart failure, hospitalization, or death from heart failure in bardoxolone methyl-treated patients in the BEACON trial was attributed to kidney-specific suppression of the endothelin pathway resulting in sodium and volume retention [19]. Given the fact that ET-1 directly decreases microvascular permeability, the inhibition of the ET-1-pathway in microvascular endothelium could increase endothelial permeability. Indeed, in a set of *ex vivo* experiments, ET-1 decreased permeability in rat mesenteric microvessels [50, 51]. Furthermore, decreased microvascular permeability caused by ET-1 was suggested to be mediated by the ET_B_ receptor [50, 51]. These reports uncovered, for the first time, an important role of ET-1 in the maintenance and modulation of endothelial permeability. Recently, Kansanen et al. [52] demonstrated that in human aortic endothelial and human umbilical endothelial cells, nitro-oleic acid, *via* an Nrf2-dependent pathway, leads to an increased expression of the ET_B_ receptor and a subsequent decrease in extracellular ET-1 secreted by endothelial cells. The authors postulated that this mechanism may limit the vasoconstrictive effects of ET-1 and could prove therapeutically useful, for example, in pulmonary artery hypertension [52]. Our results clearly indicate that ET-1 (in addition to its well-known vasoconstrictive, mitogenic, and proinflammatory effects via ET_A_ receptors, the NO- and PGI2-releasing effect via the ET_B_ receptor), quite surprisingly, is also involved in maintaining the endothelial barrier function in human microvascular endothelial cells. In fact, exogenous ET-1 increased endothelial barrier function. Our experiments are not conclusive regarding the type of ET-1 receptor involved in the regulation of endothelial permeability by ET-1, especially given a possible heterodimerization of receptor A and B subunits [53]. However, the results presented in the present work fully support the notion of ET-1 as a local autocrine regulator of endothelial barrier function, as suggested previously [50, 51, 54]. Even though downregulation of ET-1 by laminar flow has vasoprotective effects and the suppression of ET-1-dependent mechanisms by Nrf2 activators may be efficacious in the treatment of pulmonary arterial hypertension (PAH) [33, 55], suppression of local ET-1 production in the microcirculation may lead to increased endothelial permeability.

In the present work, we demonstrate that bardoxolone methyl suppressed ET-1 release from HMECs and increased microvascular endothelial permeability. These effects were seen even after 3 hours of incubation with bardoxolone methyl, an experimental setting that was not associated with endothelial toxicity (Figure 3 and Table 1) but clearly lowered ET-1 production. Dimethyl fumarate did not disturb endothelial barrier function after 3 hours of incubation, and L-sulforaphane at the highest concentrations had only minor effects that were also present after 24 hours of incubation, but again they were weaker as compared with bardoxolone methyl.

Analysis of nuclear translocation of Nrf2 revealed that in the tested concentration range of Nrf2 activators, bardoxolone methyl was the most potent inducer of the Nrf2 pathway in HMECs, while L-sulforaphane was less potent. These results are compatible with the weaker effects of L-sulforaphane on ET-1 release and endothelial permeability. Lack of significant effects of dimethyl fumarate could be attributed to the weakest effects of this compound on Nrf2 among all three Nrf2 activators in the tested concentration range. Indeed, L-sulforaphane was also shown by other authors to be a more potent inducer of Nrf2 compared with dimethyl fumarate [56]. It may also be that concomitant activation of other endothelial protective mechanisms by increasing mitochondrial respiration (as evidenced in our experiments) could play a role in a differential response of cells to dimethyl fumarate vs. L-sulforaphane [57]. Obviously, in order to identify whether the effects of bardoxolone methyl on ET-1 release and endothelial permeability are indeed mediated by Nrf2, further experiments are needed, e.g., with Nrf2 silencing.

Altogether, our results provide a novel insight into a possible detrimental influence of bardoxolone methyl on microvascular endothelium that could have contributed to the side effects of this compound reported in the BEACON study. However, the concentration range used here was higher than the therapeutic range of concentration for bardoxolone methyl [8]; thus, this conclusion needs further verification in experimental studies, best to be performed in *in vivo* experimental conditions.

In clinical trials comprising bardoxolone methyl, dimethyl fumarate, or L-sulforaphane, *C*_max_ plasma levels found in patients were 24.7 ± 13.3 ng/ml (49 nM), 1.87 mg/l (13 *μ*M), and 36.7 ng/ml (210 nM), respectively [8, 10, 58]. These results suggest that for bardoxolone methyl and L-sulforaphane, nanomolar ranges of concentrations are close to plasma concentration in patients, while it requires micromolar concentrations for dimethyl fumarate. Still, we cannot exclude that chronic treatment with bardoxolone methyl *in vivo*, in particular in patients with preexisting alterations of microvascular endothelial barrier function, e.g., due to diabetes, would result in the effects described *in vitro* using low micromolar concentrations of this compound.

In conclusion, despite the limitations described above, to the best of our knowledge, this study is the first to comprehensively evaluate the influence of three major Nrf2 activators on human microvascular endothelium to identify an endothelium-oriented explanation for the side effects of bardoxolone methyl reported in the BEACON clinical trial. We have demonstrated that bardoxolone methyl displays a distinct profile of activity in the endothelium, including detrimental effects on mitochondria and cellular viability and profound suppression of endothelial ET-1 release that could possibly interfere with ET-1-dependent autocrine regulation of endothelial permeability, safeguarding microvascular function.

## Figures and Tables

**Table 1:** Quantification of apoptosis and necrosis with the use of an Annexin V/propidium iodide assay for flow cytometry in HMEC-1 cells after a 24-hour-long incubation with bardoxolone methyl, dimethyl fumarate, and L-sulforaphane. Values are expressed as mean % of cell population ± SEM, *n* = 4. The significance of the differences between the means was evaluated by one-way analysis of variance (ANOVA) with Dunn's post hoc test if appropriate; ^∗^*p* < 0.05 and ^∗∗^*p* < 0.01 indicate significant difference vs. the control group (DMSO 0.05%). The results were obtained in four independent experiments.

Population (mean %)	Bardoxolone methyl				Dimethyl fumarate				L-sulforaphane			
	Necrosis (AV-/PI+)	Apoptosis+necrosis (AV+/PI+)	Live (AV-/PI-)	Apoptosis (AV+/PI-)	Necrosis (AV-/PI+)	Apoptosis+necrosis (AV+/PI+)	Live (AV-/PI-)	Apoptosis (AV+/PI-)	Necrosis (AV-/PI+)	Apoptosis+necrosis (AV+/PI+)	Live (AV-/PI-)	Apoptosis (AV+/PI-)
DMSO 0.05%	0.45 ± 0.03	1.15 ± 0.14	90.48 ± 0.54	7.93 ± 0.54	0.45 ± 0.03	1.15 ± 0.14	90.48 ± 0.54	7.93 ± 0.54	0.45 ± 0.03	1.15 ± 0.14	90.48 ± 0.54	7.93 ± 0.54
100 nM	1.33 ± 0.39	4.25 ± 1.71	87.98 ± 1.28	6.43 ± 1.96	2.8 ± 0.80	8.6 ± 0.80	84.75 ± 0.05	3.8 ± 0.00	0.90 ± 0.12	3.98 ± 0.48	89.80 ± 0.29	5.85 ± 0.40
300 nM	1.40 ± 0.55	4.43 ± 2.18	87.35 ± 1.43	6.85 ± 1.86	2.85 ± 0.75	7.3 ± 2.30	86.4 ± 3.50	3.4 ± 0.50	0.95 ± 0.18	3.42 ± 0.44	88.60 ± 0.65	6.47 ± 0.68
500 nM	1.40 ± 0.61	4.40 ± 2.20	86.10 ± 1.38	8.00 ± 2.49	1.75 ± 0.45	7.7 ± 1.20	86.65 ± 1.75	3.9 ± 0.20	1.10 ± 0.25	3.43 ± 0.47	89.10 ± 0.45	6.37 ± 0.55
1 *μ*M	2.68 ± 1.00	8.45 ± 2.83	83.23 ± 5.95	6.03 ± 0.85	2.4 ± 0.80	9.45 ± 2.95	84.15 ± 4.35	3.95 ± 0.65	1.05 ± 1.12	4.32 ± 0.38	88.13 ± 0.24	6.52 ± 0.45
3 *μ*M	11.33 ± 4.82^∗^	39.80 ± 11.52^∗^	33.53 ± 5.64^∗^	15.35 ± 3.37	2.7 ± 0.50	7.3 ± 1.30	86.1 ± 2.40	3.8 ± 0.60	1.85 ± 0.29	6.38 ± 1.3	83.32 ± 2.22	8.42 ± 0.78
5 *μ*M	18.90 ± 7.86^∗∗^	51.18 ± 11.80^∗∗^	17.18 ± 4.58^∗∗^	12.85 ± 3.87	2.75 ± 0.45	7.65 ± 2.25	85.7 ± 3.10	3.95 ± 0.45	1.20 ± 0.17	4.25 ± 0.55	87.23 ± 0.46	7.30 ± 1.69

## Data Availability

Answer: yes. Comment: the data is being stored on the internal servers of the Jagiellonian Centre for Experimental Therapeutics JCET UJ in Jagiellonian University of Krakow. Any person interested in viewing the data is welcome to contact either the first or the corresponding author.

## Abbreviations

ROS: Reactive oxygen species
CDDO-Me: Bardoxolone methyl
CKD: Chronic kidney disease
ET-1: Endothelin 1
ET_A_ receptor: Endothelin receptor type A
ET_B_ receptor: Endothelin receptor type B
ESRD: End-stage renal disease
HMEC-1: Human microvascular endothelial cells
MTS: 3-(4,5-Dimethylthiazol-2-yl)-5-(3-carboxymethoxyphenyl)-2-(4-sulfophenyl)-2H-tetrazolium
Nrf2: Nuclear factor (erythroid 2-related factor) 2
PGI2: Prostacyclin
PPAR-*γ*: Peroxisome proliferator-activated receptor-*γ*.
